# Costs of Cancer Prevention: Physical and Psychosocial Sequelae of Risk-Reducing Total Gastrectomy

**DOI:** 10.1200/JCO.23.01238

**Published:** 2023-10-30

**Authors:** Amber F. Gallanis, Lauren A. Gamble, Sarah G. Samaranayake, Rachael Lopez, Amanda Rhodes, Suraj Rajasimhan, Grace-Ann Fasaye, Olvan Juma, Maureen Connolly, Stacy Joyce, Ann Berger, Theo Heller, Andrew M. Blakely, Jonathan M. Hernandez, Jeremy L. Davis

**Affiliations:** ^1^ Center for Cancer Research, National Cancer Institute, National Institutes of Health, Bethesda, MD; ^2^ Clinical Center Nutrition Department, National Institutes of Health, Bethesda, MD; ^3^ Pharmacy Department, National Institutes of Health Clinical Center, Bethesda, MD; ^4^ ASRC Federal, Bethesda, MD; ^5^ Clincal Center Nursing Department, National Institutes of Health, Bethesda, MD; ^6^ Pain and Palliative Care Service, Clinical Center, National Institutes of Health, Bethesda, MD; ^7^ National Institute of Diabetes and Digestive and Kidney Diseases, National Institutes of Health, Bethesda, MD

## Abstract

**PURPOSE:**

Risk-reducing surgery for cancer prevention in solid tumors is a pressing clinical topic because of the increasing availability of germline genetic testing. We examined the short- and long-term outcomes of risk-reducing total gastrectomy (RRTG) and its lesser-known impacts on health-related quality of life (QOL) in individuals with hereditary diffuse gastric cancer syndrome.

**METHODS:**

Individuals who underwent RRTG as part of a single-institution natural history study of hereditary gastric cancers were examined. Clinicopathologic details, acute and chronic operative morbidity, and health-related QOL were assessed. Validated questionnaires were used to determine QOL scores and psycho-social-spiritual measures of healing.

**RESULTS:**

One hundred twenty-six individuals underwent RRTG because of a pathogenic or likely pathogenic germline *CDH1* variant between October 2017 and December 2021. Most patients (87.3%; 110/126) had pT1aN0 gastric carcinoma with signet ring cell features on final pathology. Acute (<30 days) postoperative major morbidity was low (5.6%; 7/126) and nearly all patients (98.4%) lost weight after total gastrectomy. At 2 years after gastrectomy, 94% (64/68) of patients exhibited at least one chronic complication (ie, bile reflux, dysphagia, and micronutrient deficiency). Occupation change (23.5%), divorce (3%), and alcohol dependence (1.5%) were life-altering consequences attributed to total gastrectomy by some patients. In patients with a median follow-up of 24 months, QOL scores decreased at 1 month after gastrectomy and returned to baseline by 6-12 months.

**CONCLUSION:**

RRTG is associated with life-changing adverse events that should be discussed when counseling patients with *CDH1* variants about gastric cancer prevention. The risks of cancer-prevention surgery should not only be judged in the context of likelihood of death due to disease if left untreated, but also based on the real consequences of organ removal.

## INTRODUCTION

Prophylactic surgery is a cancer-prevention strategy applied to individuals at risk for developing solid tumors because of pathogenic or likely pathogenic (P/LP) germline gene variants. It is necessary not only to understand surgical risks and alternatives to surgery, such as enhanced surveillance, but also to incorporate lifetime cancer risk estimates when developing a personalized management strategy. A classic example of risk-reducing surgery is familial adenomatous polyposis (FAP), which is driven by germline *APC* variants and can result in hundreds to thousands of polyps throughout the colon and rectum.^1^ The average age of colon cancer diagnosis is 39 years, with near-complete penetrance, for which colectomy with or without proctectomy is the recommended risk-reduction strategy.^2-4^ In recent decades, the rise in clinical human genomic sequencing has expanded the application of prophylactic surgery, most notably for inherited breast cancer risk.^5-7^ Specifically, identification of *BRCA1*/*BRCA2* variants through commercially available testing has been associated with a substantial increase in the rate of risk-reducing mastectomy in the United States.^8,9^

**CONTEXT**

**Key Objective**
To identify the physical and psychosocial sequelae of risk-reducing total gastrectomy (RRTG) in patients with germline *CDH1* variants and hereditary diffuse gastric cancer syndrome.
**Knowledge Generated**
At 2 years after RRTG, nearly all patients experienced at least one chronic morbidity such as bile reflux, dysphagia, micronutrient deficiency, or need for supplemental enteral tube feeding. Given the life-altering consequences of surgery for the prevention of cancer, the benefits of RRTG must be considered along with the chronic physical and psychosocial costs to patients.
**Relevance *(E.M. O'Reilly)***
The manuscript provides insights into the outcomes beyond oncologic considerations. It is a reminder of the importance of both short- and long-term sequelae following a risk reduction intervention, such as gastrectomy, and the not insignificant medical and other concerns.*
  *Relevance section written by *JCO* Associate Editor Eileen M. O'Reilly, MD.

Clinical decision making in at-risk populations incorporates knowledge of cancer risk and the options for prevention or risk reduction in an individual. When surgery to remove an at-risk organ is considered, the potential impact on overall health and well-being must also be considered. Quality-of-life (QOL) changes associated with risk-reducing surgery in patients with *BRCA1*/*BRCA2* and other breast cancer predisposition gene variants have been well described.^10-12^ For example, multiple studies have demonstrated that body image and sexual well-being were negatively affected after mastectomy.^11,12^ Similarly, the clinical impact of prophylactic proctocolectomy in young adults with FAP has prompted discussion about less extensive surgery with indefinite cancer surveillance, and the lifelong impacts of reconstructive intestinal surgery.^13-15^ However, the physical and psychosocial impacts of risk-reducing surgery for other solid tumors is not well known.

Loss-of-function mutations in the tumor suppressor gene *CDH1* are causally linked to hereditary diffuse gastric cancer (HDGC) and hereditary lobular breast cancer,^16,17^ making this a unique cohort for which there are potentially two indications for risk-reducing surgery. A minority of HDGC cases are attributed to germline variants in *CTNNA1*.^18,19^ Risk-reducing total gastrectomy (RRTG) is recommended to germline *CDH1* P/LP variant carriers because the estimated lifetime risk of diffuse-type gastric cancer is 25%-42%, with some population estimates as high as 80%.^20,21^ Although recent studies have demonstrated that endoscopic surveillance with random and targeted biopsies can be a reasonable alternative to surgery in some patients, the long-term safety of surveillance is not yet known.^22,23^ Attempts to risk-stratify individuals on the basis of genotype or family cancer history to help guide clinical management have not been successful.^24,25^

Total gastrectomy is an uncommon operation with a plethora of potential acute and chronic complications.^26-28^ Although short-term operative risks and early recovery patterns have been described, the long-term consequences of total gastrectomy remain ill defined. In healthy individuals with germline *CDH1* variants and an otherwise normal life expectancy, the best estimates of individuals' lifetime risks of HDGC versus the long-term QOL outcomes and psychosocial implications of total gastrectomy are critical to consider to inform preoperative decision making most appropriately. We sought to examine the physical and psychosocial impacts of RRTG in individuals with *CDH1* P/LP variants. With a focus on short- and long-term outcomes that encompass both physical health and QOL, we aimed to shed light on the diversity of personal consequences that may guide counseling for risk-reducing surgery and aid decision making.

## METHODS

Individuals enrolled in a natural history study of hereditary gastric cancers (ClinicalTrials.gov identifier: NCT03030404) and who harbored a *CDH1* P/LP germline variant were eligible for this retrospective analysis. Patients with *CTNNA1* variants were excluded. All individuals received counseling for management of gastric cancer risk, including international consensus recommendations for RRTG and the option for annual endoscopic surveillance for those who declined, delayed, or were medically unfit for surgery.^18^ Individuals who elected for RRTG underwent a standardized preoperative evaluation by a registered dietitian, a clinical pharmacist, a gastroenterologist, and a surgical oncologist. A licensed clinical social worker evaluated patient employment/insurance status, mental health history, alcohol and drug use, and presence of social support system. RRTG was ascribed to individuals without gross findings of gastric cancer at the time of screening esophagogastroduodenoscopy. Operations were standardized and performed by the same surgeon (J.L.D.) with a D1 (perigastric) lymphadenectomy and Roux-en-Y esophagojejunostomy using a 50-cm retro-colic Roux limb. Postoperative adverse events were recorded according to the classification by Dindo et al.^29^ Acute morbidity was recorded at 30 days postoperatively and throughout the follow-up period. Postgastrectomy clinical evaluations and nutritional assessments were performed at 1, 3, 6, and 12 months postoperatively and annually thereafter.

Comprehensive clinical questionnaires were administered to individuals with a minimum of 2 years of postgastrectomy follow-up. The National Institutes of Health Healing Experience of All Life Stressors (NIH-HEALS) questionnaire was completed by patients before surgery and at 1, 3, 6, 12, and 24 months after RRTG to assess psycho-social-spiritual healing.^30^ Subscores were calculated as previously described.^30^ The Functional Assessment of Cancer Therapy-General (FACT-G) and Gastric (FACT-Ga) questionnaires were used to assess QOL after RRTG.^31^ QOL measures were determined by calculating physical, social, emotional, and functional well-being scores, and a gastric cancer–specific subscore. Three scores were calculated: FACT-Gastric Trial Outcome Index (TOI), FACT-G, and FACT-Ga as previously described.^31,32^ We analyzed data from patients who completed baseline surveys and at least one postgastrectomy time point, and excluded those without baseline surveys. One-way analyses of variance were performed to compare scores at baseline and postgastrectomy time points using GraphPad Prism Version 9.3.1 (GraphPad Software, Inc, San Diego, CA). All research-related clinical care was provided at NIH Clinical Center, Bethesda, MD. This study was approved by the institutional review board of the NIH, and all patients provided informed written consent.

## RESULTS

Physical and psychosocial outcomes in 126 consecutive individuals undergoing RRTG between October 2017 and December 2021 were analyzed. Most individuals were female (75%) and White (97%) and had a median age of 43 years (range, 19-71) at operation (Table 1). The geographically and genetically heterogeneous cohort consisted of 79 distinct families with primary residence throughout the United States. Nearly all patients (96%; 121/126) had a family history of gastric cancer and 67.5% (85/126) had a family history of breast cancer. Family or personal history of cleft lip/palate, which is part of the HDGC syndrome due to *CDH1*, was reported by 12.7% (16/126) of patients. The most common *CDH1* variant among individuals were splicing variants (43.7%, 55/126), followed by nonsense (25.4%, 32/126) and frameshift variants (23%, 29/126; Appendix Table A1, online only). Seven patients exhibited a large gene deletion, two had a start-loss variant, and one had a large duplication variant.

**TABLE 1.** Patient Demographics

Patient Demographic	N = 126
Sex, No. (%)	
Female	95 (75)
Male	31 (25)
Families, No.	79
Age, years, median (range)	43 (19-71)
Race, No. (%)	
White	122 (97)
Black or African American	3 (2)
Hispanic	1 (1)
Genotype, No. (%)	
Variant	
Splicing	55 (43.7)
Nonsense	32 (25.4)
Frameshift	29 (23.0)
Large deletion	7 (5.6)
Start-loss	2 (1.6)
Large duplication	1 (0.8)
Family history, No. (%)	
Gastric cancer	121 (96)
Breast cancer	85 (67.5)
Cleft lip/palate	16 (12.7)
Baseline EGD with SRC, No. (%)	59 (47.2)
Surgical approach, No. (%)	
Open	124 (98.4)
Robotic-assisted	2 (1.6)
Secondary procedure performed, No. (%)	27 (21.4)
Cholecystectomy	23 (18.3)
Salpingo-oophorectomy	2 (1.6)
Umbilical hernia repair	1 (0.8)
Splenectomy	1 (0.8)
Operative time, minutes, median (range)	168 (111-373)
Estimated blood loss, mL, median (range)	50 (5-600)
Postoperative ICU admission, No. (%)	6 (4.8)
Epidural used, No. (%)	125 (99.2)
Final pathology on gastrectomy specimen, No. (%)	
ypT1a	110 (87.3)
ypTis	6 (4.8)
ypT0	10 (7.9)
ypN0	126 (100)

Abbreviations: EGD, esophagogastroduodenoscopy; ICU, intensive care unit; SRC, signet ring cell.

Upper endoscopy was performed before surgery in all patients except one who previously underwent Roux-en-Y gastric bypass (RYGB). Random endoscopic gastric biopsies revealed occult, microscopic foci of signet ring cell carcinomas in approximately half (47.2%, 59/125) of the patients, which is consistent with the disease phenotype.^22,23^ Total gastrectomy was conducted with an open surgical approach in all but two cases. Median operative duration was 168 minutes (range, 111-373 minutes) with a median estimated blood loss of 50 mL. A concurrent procedure (eg, cholecystectomy) was performed in 21.4% of cases. Gross pathologic examination revealed normal-appearing gastric mucosa in all patients. Pathologic diagnosis of T1aN0 gastric carcinoma with signet ring cell features was made in 87.3% (110/126) of patients, whereas 4.8% (6/126) and 7.9% (10/126) had T*is*N0 and T0N0 pathology, respectively (Table 1). There were no cancer recurrences or cancer-related deaths during the follow-up period.

### Acute Sequelae of RRTG

The rate of overall major postoperative complications (Clavien-Dindo grade ≥3) was 18.3% (23/126) and there was no postoperative mortality. The most frequent complications were attributed to esophagojejunostomy anastomotic leak. Hospital readmission rate within 30 days postoperatively was 7.1% (9/126; Table 2). Reoperation or procedural intervention within 30 days occurred in 5.6% (7/126), with a median of 7 days (range, 4-27) to reintervention (Table 2). Indications for reintervention were anastomotic leak, bleeding, and intra-abdominal abscess drainage. Another patient required splenic artery embolization and subsequent hematoma evacuation after likely splenic artery pseudoaneurysm rupture after drain placement for an intra-abdominal fluid collection.

**TABLE 2.** Complications After RRTG

Event	N = 126
Reoperation/intervention within 30 days, No. (%)	7 (5.6)
Time, days, median (range)	7 (4-27)
Hospital readmissions within 30 days, No. (%)	9 (7.1)
Anastomotic leak	2 (1.6)
Pneumonia	3 (2.4)
Wound infection	2^a^ (1.6)
Pancreatitis	2 (1.6)
Duodenal stump leak	1 (0.8)
Surgical site infection, No. (%)	4 (3.2)
Anastomotic leak, esophagus, No. (%)	8 (6.3)
Postoperative day diagnosed, median (range)	7 (4-14)
Esophageal stent placement, No. (%)	8 (100)
Duodenal stump leak, No. (%)	3 (2.4)
Anastomotic stricture, No. (%)	2 (1.6)
Enteral feeding tube placement, No. (%)	11 (8.7)
Failure to thrive, No.	5
Leak, No.	5
Dehydration, No.	1
Postoperative feeding tube placed, days, median (range)	67 (9-491)
Incisional hernia, No. (%)	14 (11.1)
Small bowel obstruction, No. (%)	1 (0.8)
Symptomatic cholelithiasis, No. (%)	6 (4.8)
Nephrolithiasis, No. (%)	9 (7.1)

Abbreviation: RRTG, risk-reducing total gastrectomy.

^a^ One patient with duodenal stump leak and wound infection.

### Weight Loss After RRTG

**FIG 1.**
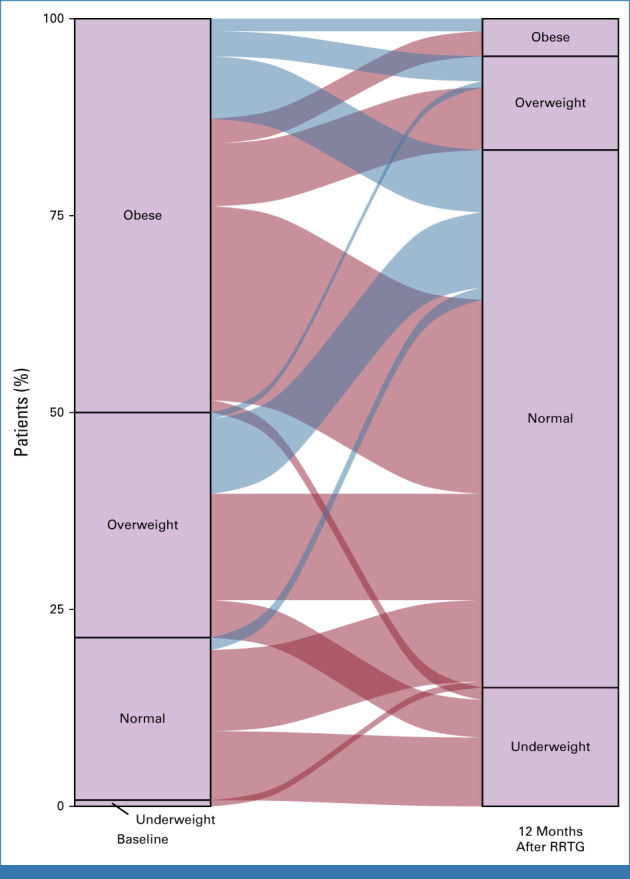
Change in BMI from baseline to 12 months after RRTG in 126 patients by sex (males = blue, females = red). Underweight BMI is defined as ≤18.4 kg/m^2^, normal BMI is 18.5-24.9 kg/m^2^, overweight BMI is 25-29.9 kg/m^2^, and obese BMI is >30 kg/m^2^. RRTG, risk-reducing total gastrectomy.

Weight loss is a sine qua non of total gastrectomy because of the restrictive and malabsorptive nature of the operation. Nearly all patients (98.4%; 124/126) registered below their preoperative weight at 12 months after gastrectomy with a median BMI change of –8.1 kg/m^2^ (IQR, –11.3 to –5.3 kg/m^2^). At 12 months after gastrectomy, most patients (68.3%; 86/126) had a normal BMI (18.5-24.9 kg/m^2^), whereas 15.1% (19/126) were underweight (BMI ≤18.4 mg/kg^2^), 11.1% (14/126) were overweight (BMI 25-29.9 mg/kg^2^), and 5.6% (7/126) were obese (BMI >30 mg/kg^2^; Fig 1). Feeding jejunostomy tube placement for enteral feeding was instituted in 11 patients (8.7%; 11/126) at some time during the follow-up period. Indications for feeding tubes included failure to thrive (n = 5), esophageal anastomotic leak (n = 5), and persistent dehydration (n = 1).

### Chronic Sequelae of RRTG

**FIG 2.**
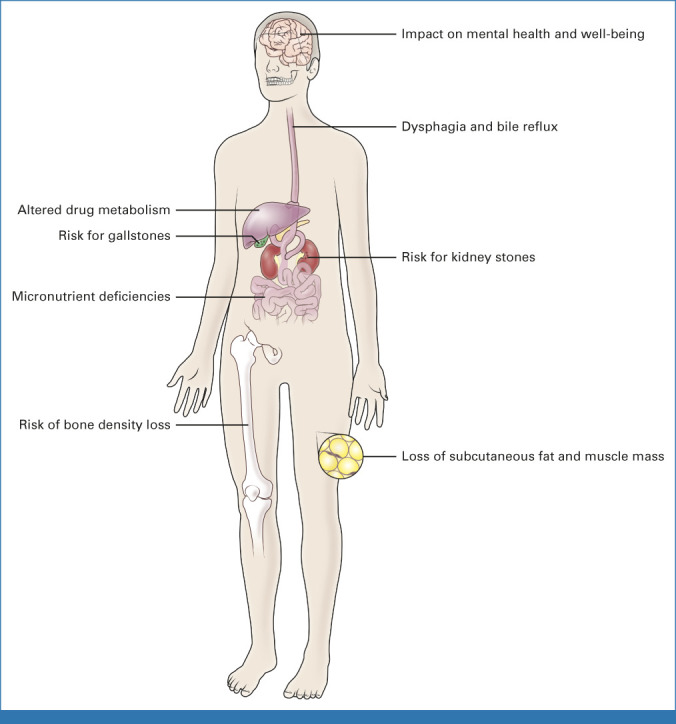
Schematic demonstrating the chronic sequelae and impact on multiple organ systems after risk-reducing total gastrectomy.

To better understand the long-term consequences of total gastrectomy, we analyzed patients with a minimum follow-up of 24 months after gastrectomy (n = 68) with the aid of a comprehensive clinical questionnaire. Overall, the physical and psychosocial sequelae of RRTG were pervasive and affected multiple organ systems, such that 94% (64/68) of patients exhibited at least one chronic complication such as bile reflux, dysphagia, or micronutrient deficiency (Fig 2).

### Postgastrectomy Nutrition and Micronutrient Supplementation

All patients received preoperative and postoperative education on the lifelong need for daily oral micronutrient supplementation with commercially available bariatric multivitamins and calcium citrate. Peripheral blood micronutrient levels were assessed at regularly scheduled clinical follow-up. The most common micronutrient deficiencies after RRTG were of iron, vitamin D, vitamin B_12_, thiamine, and folate. Intravenous or intramuscular micronutrient correction was based on abnormal laboratory values; 29.4% (20/68) received at least one iron infusion, 16.2% (11/68) received a vitamin B_12_ injection, 7.4% (5/68) received a thiamine infusion, and 2.9% (2/68) received a folate injection (Table 3).

**TABLE 3.** Chronic Sequelae of RRTG

Event	N = 68, No. (%)
Micronutrient deficiencies requiring infusion	
Iron	20 (29.4)
Vitamin B12	11 (16.2)
Thiamine	5 (7.4)
Folate	2 (2.9)
Bile reflux	50 (73.5)
Mild	17 (25)
Moderate	16 (23.5)
Severe	13 (19.1)
Very severe	4 (5.9)
Dysphagia	10 (14.7)
Feeling of food stuck in the esophagus	15 (22.1)
Mental illness	
Preexisting GAD, depression, BPD	15 (22.1)
New-onset GAD, depression	5 (7.4)
Employment change	16 (23.5)
Dumping syndrome	n = 50
Early dumping syndrome	16 (32)
Late dumping syndrome	22 (44)

Abbreviations: BPD, bipolar disorder; GAD, generalized anxiety disorder; RRTG, risk-reducing total gastrectomy.

### Postgastrectomy GI Symptoms

Many of the classic postgastrectomy syndromes are the consequence of stomach removal and loss of the antireflux mechanism, food storage capacity, and hormone signaling.^33^ Bile reflux was the most pervasive GI symptom, reported by 73.5% (50/68) of patients with a minimum of 2 years of follow-up (Table 3). Bile reflux was classified as mild, moderate, severe, or very severe.^34^ Twenty-five percent (17/68) of patients reported mild bile reflux that was managed with diet and lifestyle modifications (ie, remaining upright after eating and sleeping on a wedge pillow) and over-the-counter (OTC) medications such as calcium carbonate and sodium alginate-bicarbonate. Patients with moderate reflux symptoms that persisted after lifestyle modifications/OTC medication (23.5%; 16/68) were trialed with prescription medication (eg, cholestyramine). Severe or very severe bile reflux that interfered with activities of daily living occurred in 19.1% (13/68) and 5.9% (4/68) of patients, respectively, and prompted clinical evaluation with endoscopy. One patient had erosive esophagitis (LA grade D)^35^ and subsequently underwent Roux limb lengthening with resolution of symptoms.

Reports of dysphagia after total gastrectomy warrant clinical evaluation to rule out mechanical causes such as anastomotic stricture. Fifteen (22.1%) of 68 patients reported feeling of food stuck in the esophagus. Two of 15 patients had an anastomotic stricture of the esophagojejunostomy site that required dilation. Other cases of difficulty swallowing resulted in diagnosis of diffuse esophageal spasm in one patient and esophageal dysmotility in four patients. Dysphagia attributed to esophageal dysmotility was treated with promotility medications in 19.1% (13/68) of patients with variable clinical success. Finally, symptom documentation on dumping syndrome was available in 50 patients. Episodic early- and late-dumping syndrome was reported in 32% (16/50) and 44% (22/50) of patients, respectively (Table 3). Dumping syndrome was most often remedied through intensive nutrition education and frequent counseling by a registered dietitian.

### Postgastrectomy Abdominal Pain

Abdominal pain due to nephrolithiasis was diagnosed in 7.1% (9/126) of patients, one of whom required urologic intervention. Although stone analysis was not performed, RYGB has been linked to increased calcium oxalate stone formation and risk of cholelithiasis due to rapid weight loss.^36-38^ Six (4.8%) of 126 patients developed symptomatic cholelithiasis and five subsequently underwent cholecystectomy. Other causes of recalcitrant abdominal pain included small intestinal bacterial overgrowth in two patients, small bowel obstruction due to internal hernia in one patient 3.5 months after gastrectomy, and one patient who underwent exploratory laparoscopy for recurrent abdominal pain and had multiple sites of incidental small bowel intussusception. Of all 126 patients, 14 patients (11.1%) developed incisional hernias postoperatively, 13 of whom underwent operative repair.

### Psychosocial Sequelae of RRTG

RRTG imparts lifestyle changes and psychosocial costs that are not well characterized. In patients with a minimum of 24 months of follow-up, 22% (15/68) reported a preoperative diagnosis of generalized anxiety disorder, depression, or bipolar disorder. Anxiety specifically related to the diagnosis of *CDH1* P/LP variant was self-reported in four patients. Five patients reported a new diagnosis of depression or anxiety after total gastrectomy. Fifteen (75%; 15/20) were managed with prescription medications. Diagnosis of a P/LP gene mutation can affect family dynamics, employment, insurability, and interpersonal relationships.^39^ After total gastrectomy, many patients (23.5%; 16/68) reported an employment change during the follow-up period. Occupational changes were often attributed to the inability to perform work because of persistent GI symptoms after gastrectomy, such as nausea, fatigue, and inability to tolerate oral intake or eat frequent meals while working. Two patients changed jobs to have better access to medical care and health insurance to meet medical needs after gastrectomy. One patient developed alcohol dependence requiring inpatient rehabilitation. Two patients divorced and four patients married after RRTG. Additionally, four women (of whom one used in vitro fertilization with preimplantation genetic testing) gave birth to healthy children and one woman had a miscarriage postoperatively.

### QOL After RRTG

**FIG 3.**
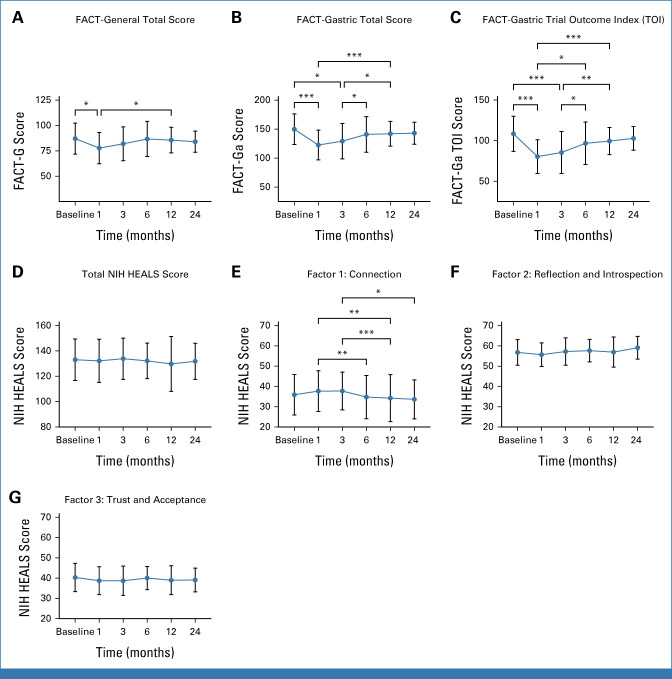
FACT-G and FACT-Ga questionnaires included 27 and 46 items, respectively, scored on a five-point Likert scale from not at all (0) to very much (4). The FACT-Ga TOI was calculated by adding physical and functional well-being scores. The FACT-G total score included physical, social, emotional, and functional well-being scores. The FACT-Ga total score combined the FACT-G score with a 19-item gastric cancer subscale. Examples of physical, social, emotional, and functional well-being items were “I have a lack of energy,” “I get emotional support from my family,” “I worry that my condition will get worse,” and “I am able to enjoy life,” respectively. The 19-item gastric cancer subscale score included disease-specific questions such as “I am bothered by reflux or heartburn” and “My digestive problems interfere with my usual activities.” (A) FACT-G, (B) FACT-Ga, and (C) TOI scores were calculated in 54 patients with germline *CDH1* variants at baseline and 1, 3, 6, 12, and 24 months after risk-reducing total gastrectomy. NIH-HEALS questionnaire included 35 items scored on a five-point Likert scale from strongly disagree (1) to strongly agree (5) with four items reverse scored (6, 23, 28, and 34). (D) Total score and subscores (E) connection to a higher power, community, and family, (F) reflection and introspection or the ability to find meaning and purpose in activities that connect mind and body, and (G) trust and acceptance that caregivers, friends, and family will respond when needs arise were calculated. Examples of questionnaire items included “My situation strengthened my connection to a higher power” for the connection factor, “I gain awareness from self-reflection” for the reflection and introspection factor, and “I am content with my life” for the trust and acceptance factor. (D) Total score and (E-G) subscores were calculated in 54 patients with germline *CDH1* variants at baseline and 1, 3, 6, 12, and 24 months after risk-reducing total gastrectomy. *P ≤ .05, **P ≤ .01, ***P ≤ .001. FACT-G, Functional Assessment of Cancer Therapy General; FACT-Ga, Functional Assessment of Cancer Therapy-Gastric; NIH-HEALS, National Institutes of Health Healing Experience of All Life Stressors; TOI, Trial Outcome Index.

QOL measures decreased across all FACT scores at 1 month after gastrectomy (Fig 3). Although FACT-G scores decreased at 1 month after RRTG (*P* = .03), by 12 months, QOL scores had increased (*P* = .02; Fig 3A). There were no differences in FACT-G scores from baseline to 3, 6, 12, and 24 months postoperatively (Fig 3A). Compared with baseline, total FACT-Ga scores decreased at 1 and 3 months after gastrectomy (*P* < .001 and *P* = .01, respectively). However, FACT-Ga QOL scores increased from 1 month to 12 months (*P* < .001), 3 months to 6 months (*P* = .04), and 3 months to 12 months (*P* = .02) after surgery (Fig 3B). FACT-Ga TOI, a marker of functional status, also decreased significantly from baseline to 1 and 3 months postoperatively (*P* < .001; Fig 3C). Interestingly, we found functional status measures increased from 1 month to 6 months (*P* = .05) and 1 month to 12 months (*P* < .001) after gastrectomy. Before RRTG, 54 patients completed the NIH-HEALS and FACT-G/Ga surveys and 36, 32, 21, 27, and 11 patients completed the survey at 1, 3, 6, 12, and 24 months, respectively. There was no difference in the overall NIH-HEALS score at baseline compared with 1, 3, 6, 12-,and 24 months after gastrectomy (Fig 3D). NIH-HEALS Connection scores decreased from 1 month to 6 months (*P* = .004) and from 1 month to 12 months (*P* = .001) after gastrectomy (Fig 3E); however, patient-reported domains of reflection and introspection and trust and acceptance did not change over time (Figs 3F and 3G).

## DISCUSSION

Hereditary cancer syndromes present unique opportunities for cancer prevention and challenging decisions for health care providers and patients alike. We elucidated the burden of total gastrectomy for prevention of advanced gastric cancer in individuals with *CDH1* P/LP variants. Although occult, early-stage signet ring cell carcinoma is a common finding at RRTG, additional research is needed to elucidate the mechanisms by which some of these lesions will progress to advanced cancer and others will not.^40,41^ Until accurate cancer risk stratification is available, the risk reduction achieved with RRTG is absolute, albeit consequential. Nearly all patients in this study exhibited at least one chronic sequela of RRTG. With no gastric cancer recurrence or cancer-related deaths in this cohort, we demonstrated the magnitude of acute and chronic effects of RRTG on physical and psychosocial well-being and persistent health changes in multiple body systems. Although the rate of major postoperative complications was low, which speaks to the safety of the operation, nearly all patients experienced the enduring consequences of RRTG that included micronutrient deficiencies, fatigue, dysphagia, bile reflux, and depression.

One aim of this study was to improve our understanding of postgastrectomy outcomes with validated QOL surveys. We found that physical, social, emotional, and functional well-being scores significantly decreased in the first month after surgery, then returned to baseline by 6 months, which is similar to previous QOL studies in patients undergoing TG.^42,43^ This suggests that resources should be focused on optimizing recovery in the immediate postoperative period. However, we found that QOL questionnaires did not capture many of the challenges faced by patients months after RRTG. Although Worster et al^43^ found physical and functional QOL scores returned to baseline by 12 months after gastrectomy, intrusive GI symptoms, such as diarrhea, strict dietary restrictions, and reflux persisted. Muir et al^42^ also found QOL scores decreased immediately after gastrectomy, returned to baseline by 12 months, yet declined again at 24 months. In their study, pain, fatigue, insomnia, dyspnea, and loss of appetite were the most common complaints after gastrectomy. Chronic fatigue and weight loss affecting body image have also been reported after gastrectomy.^44^ Despite potential lifelong morbidity, most patients who undergo RRTG report being satisfied with their decision.^27,45^ Although QOL assessment tools can be helpful, they are unlikely to capture the complete experience in patients undergoing RRTG. In the current study, one in four patients changed jobs after gastrectomy. Reasons for job change included inability to perform similar occupation tasks as presurgery, chronic fatigue, and persistent postoperative GI symptoms such as nausea and poor oral intake. These data are consistent with a report by Hallowell et al^44^ demonstrating that patients undergoing TG can experience negative financial consequences because of the inability to return to work or to work full time. Our findings emphasize the importance of access to medical care and health insurance for management of long-term sequelae after RRTG.

The impact of elevated cancer risk because of germline gene mutations and recommendation for risk-reducing surgery are major stressors for individuals and families. Many patients we surveyed had a concurrent diagnosis of mental illness, with anxiety being the most common. Although we did not compare patient-reported anxiety presurgery and postsurgery, multiple studies have shown that presurgery anxiety was significantly reduced postoperatively in women at high risk for breast cancer who underwent bilateral prophylactic mastectomy (BPM).^10,46,47^ McCarthy and colleagues demonstrated that women who underwent BPM with reconstruction had higher psychosocial well-being scores, yet lower physical well-being scores of the chest and upper body, at 1 and 2 years postoperatively.^10^ BPM can also negatively affect women's self-esteem and body image, further impairing personal views about sexuality and sex life.^46,47^ Interestingly, women who underwent psychological consultation before BPM had improved psychosocial outcomes and a more positive body image.^48,49^ This underscores the importance of formal preoperative psychosocial assessment and interdisciplinary management of individuals being considered for risk-reducing surgery.

In conclusion, risk-reducing surgery for prevention of cancer is associated with a myriad of physical and psychosocial sequelae that may vary greatly by organ system. For individuals at risk for diffuse gastric cancer, an interdisciplinary clinical team is crucial to properly prepare and care for patients who elect for total gastrectomy. A thorough consideration of surgical morbidity and the purported benefits of cancer risk reduction are vital to fully inform all patients with hereditary cancer syndromes who are considering surgery. Alternatives to surgery, such as enhanced surveillance, warrant counseling about risks of a missed cancer diagnosis or development of incurable cancer. For *CDH1* variant carriers, the long-term sequelae of total gastrectomy, not just acute operative risk, should be given equal consideration as the chance of developing advanced gastric cancer.

## Data Availability

Deidentified data may be shared upon reasonable request from the corresponding author.

## APPENDIX

**TABLE A1.** Index of Germline CDH1 Variants by Patient and Corresponding Family Cancer History

Nucleotide Change (HGVS)	Patients With Variant (No.)	Families With Variant (No.)	Variant Type	ACMG/AMP CDH1 Criteria	ACMG/AMP CDH1 Classification	Family History of Gastric Cancer, No. of Patients	Family History of Breast Cancer, No. of Patients	Family History of Cleft Lip/Palate, No. of Patients
c.1008G>T	1	1	Splicing, coding	PVS1_Moderate, PM2, PS3, PS4_Supporting	PV/LPV	1	1	0
c.1064dup	1	1	Frameshift	PVS1_Very strong, PP5_Moderate, PM2_Supporting	PV	0	1	0
c.1137G>A	2	2	Splicing, coding	PVS1_Moderate, PS3, PS4, PM2	PV/LPV	2	2	1
c.1145del	1	1	Frameshift	PVS1_Very strong, PP5_Very strong, PM2_Supporting	PV	1	0	0
c.1189A>T	1	1	Nonsense	PVS1_Very strong, PP5_Strong, PM2_Supporting	PV	1	1	0
c.1342dup	1	1	Frameshift	PVS1_Very strong, PP5_Moderate, PM2_Supporting	PV	0	0	0
c.1460_1461del	2	1	Frameshift	PM5_Supporting, PVS1, PM2_Supporting	PV	2	2	0
c.1476_1477del	4	2	Frameshift	PVS1, PM2, PS4_Supporting	PV/LPV	4	2	0
c.1488_1494del	2	1	Frameshift	PVS1, PM2, PS4_Supporting	PV/LPV	2	2	0
c.1553_1565+39del	4	1	Splicing, intronic, coding	PVS1_Very strong, PP5_Very strong, PM2_Supporting	PV	4	3	0
c.1565+1G>A	7	4	Splicing, intronic	PVS1_Strong, PM2, PS3_Moderate, PS4, PP1_Strong	PV	7	5	3
c.1565+1G>C	4	2	Splicing, intronic	PVS1_Strong, PS4, PP1_Strong, PM2_Supporting	PV	4	1	2
c.1565+1G>T	2	1	Splicing, intronic	PVS1_Strong, PM2, PS4_Moderate, PP1_Moderate	PV/LPV	2	1	0
c.1565+2_1565+3insTT	2	2	Splicing, intronic	PP5_Very strong, PP3_Strong, PM2_Supporting	PV	2	1	0
c.1587dup	2	1	Frameshift	PVS1, PS4_Supporting, PM2	PV	2	2	0
c.1792C>T	7	6	Nonsense	PVS1, PS2, PS4, PM2, PP1_Strong	PV/LPV	6	5	1
c.1895_1896del	1	1	Frameshift	PVS1, PS4_Moderate, PM2	PV	1	1	0
c.1982del	1	1	Frameshift	PVS1_Very strong, PP5_Moderate, PM2_Supporting	PV	1	1	0
c.2054dup	1	1	Frameshift	PVS1, PM2, PS4_Supporting	PV	1	0	0
c.2064_2065del	21	12	Nonsense	PVS1, PM2, PS4	PV	21	17	3
c.2165-1G>C	1	1	Splicing, intronic	PVS1_Strong, PM2_Supporting, PS4_Supporting	LPV	1	1	1
c.2195G>A	6	5	Splicing, coding	PS3, PS4_Moderate, PM2, PP3	PV/LPV	6	2	0
c.220C>T	1	1	Nonsense	PVS1, PM2, PS4_Supporting	PV	1	1	0
c.2276del	2	1	Frameshift	PVS1, PM2, PS4_Supporting	PV	2	1	0
c.2287G>T	2	2	Nonsense	PVS1, PM2, PS4_Supporting	PV	2	0	0
c.2439+5_2439+8del	2	1	Splicing, intronic	PS4_Supporting, PM2, PP3	PV/LPV	2	1	0
c.261del	3	1	Frameshift	PM5_Supporting, PVS1, PM2_Supporting	PV	3	2	0
c.2T>G	2	1	Start-loss	PS4_Moderate, PVS1, PM2_Supporting	PV	2	2	0
c.377dup	1	1	Frameshift	PVS1, PM2, PS4_Supporting	PV/LPV	1	1	0
c.480_486delCATCAGCinsAGAATA	2	1	Frameshift	PVS1, PS4_Supporting, PM2	PV	2	2	1
c.49-2A>C	1	1	Splicing, intronic	PVS1_Strong, PS3_Moderate, PS4, PM2, PP1	PV	1	0	0
c.504del	2	1	Frameshift	PVS1, PM2, PS4_Supporting	PV	2	0	0
c.532-1G>C	1	1	Splicing, intronic	PVS1_Strong, PS4_Moderate, PM2	LPV	1	1	1
c.603del	2	1	Frameshift	PVS1, PM2, PS4_Supporting	PV	2	0	0
c.715G>A	10	6	Splicing, coding	PS4, PS3, PM2_Supporting, PP3	PV	10	5	0
c.720del	1	1	Frameshift	PM2, PVS1, PS4_Supporting	PV	1	1	0
c.832+1G>A	5	1	Splicing, intronic	PVS1_Strong, PS4_Supporting, PM2	PV/LPV	5	5	0
c.833-2A>G	7	3	Splicing, intronic	PVS1_Strong, PS4_Moderate, PP1_Moderate, PM2, PS3_Moderate	PV	6	5	0
Deletion exon 3	5	3	Large deletion	PVS1, PS4, PM2	PV	5	4	3
Deletion exons 1-2	2	2	Large deletion	PVS1, PM2, PS4	PV/LPV	2	2	0
Duplication exons 3-9	1	1	Large duplication	—	—	0	1	0

Abbreviations: ACMG, American College of Medical Genetics; AMP, Association for Molecular Pathology; HGVS, Human Genome Variation Society; LPV, likely pathogenic variant; PV, pathogenic variant.

## References

[1] Kanth P Grimmett J Champine M : Hereditary colorectal polyposis and cancer syndromes: A primer on diagnosis and management. Am J Gastroenterol 112:1509-1525, 2017 28786406 10.1038/ajg.2017.212

[2] Lynch HT de la Chapelle A: Hereditary colorectal cancer. N Engl J Med 348:919-932, 2003 12621137 10.1056/NEJMra012242

[3] Koskenvuo L Ryynänen H Lepistö A: Timing of prophylactic colectomy in familial adenomatous polyposis. Colorectal Dis 22:1553-1559, 2020 32441460 10.1111/codi.15151

[4] Tudyka VN Clark SK: Surgical treatment in familial adenomatous polyposis. Ann Gastroenterol 25:201-206, 2012 24714154 PMC3959379

[5] Newman B Austin MA Lee M : Inheritance of human breast cancer: Evidence for autosomal dominant transmission in high-risk families. Proc Natl Acad Sci U S A 85:3044-3048, 1988 3362861 10.1073/pnas.85.9.3044 PMC280139

[6] Berger ER Golshan M: Surgical management of hereditary breast cancer. Genes (Basel) 12:1371, 2021 34573353 10.3390/genes12091371 PMC8470490

[7] Mau C Untch M: Prophylactic surgery: For whom, when and how? Breast Care (Basel) 12:379-384, 2017 29456469 10.1159/000485830 PMC5803721

[8] Liede A Cai M Crouter TF : Risk-reducing mastectomy rates in the US: A closer examination of the Angelina Jolie effect. Breast Cancer Res Treat 171:435-442, 2018 29808287 10.1007/s10549-018-4824-9 PMC6096880

[9] Euhus DM: Risk-reducing mastectomy for BRCA gene mutation carriers. Ann Surg Oncol 22:2807-2809, 2015 25821000 10.1245/s10434-015-4537-9

[10] McCarthy CM Hamill JB Kim HM : Impact of bilateral prophylactic mastectomy and immediate reconstruction on health-related quality of life in women at high risk for breast carcinoma: Results of the mastectomy reconstruction outcomes consortium study. Ann Surg Oncol 24:2502-2508, 2017 28612125 10.1245/s10434-017-5915-2 PMC5706783

[11] Aygin D Cengiz H: Life quality of patients who underwent breast reconstruction after prophylactic mastectomy: Systematic review. Breast Cancer 25:497-505, 2018 29721811 10.1007/s12282-018-0862-8

[12] Razdan SN Patel V Jewell S : Quality of life among patients after bilateral prophylactic mastectomy: A systematic review of patient-reported outcomes. Qual Life Res 25:1409-1421, 2016 26577764 10.1007/s11136-015-1181-6 PMC4867133

[13] Yen T Stanich PP Axell L : APC-associated polyposis conditions, in Adam MP Everman DB Mirzaa GM (eds): GeneReviews, University of Washington, Seattle, 1993 20301519

[14] Menahem B Alves A Regimbeau JM : Colorectal family polyadenomatous diseases. What management in 2020? J Visc Surg 157:127-135, 2020 32113818 10.1016/j.jviscsurg.2019.12.003

[15] Stanich PP Sullivan B Kim AC : Endoscopic management and surgical considerations for familial adenomatous polyposis. Gastrointest Endosc Clin N Am 32:113-130, 2022 34798980 10.1016/j.giec.2021.08.007

[16] Guilford P Hopkins J Harraway J : E-cadherin germline mutations in familial gastric cancer. Nature 392:402-405, 1998 9537325 10.1038/32918

[17] Brooks-Wilson AR Kaurah P Suriano G : Germline E-cadherin mutations in hereditary diffuse gastric cancer: Assessment of 42 new families and review of genetic screening criteria. J Med Genet 41:508-517, 2004 15235021 10.1136/jmg.2004.018275 PMC1735838

[18] Blair VR McLeod M Carneiro F : Hereditary diffuse gastric cancer: Updated clinical practice guidelines. Lancet Oncol 21:e386-e397, 2020 32758476 10.1016/S1470-2045(20)30219-9 PMC7116190

[19] Coudert M Drouet Y Delhomelle H : First estimates of diffuse gastric cancer risks for carriers of CTNNA1 germline pathogenic variants. J Med Genet 59:1189-1195, 2022 36038258 10.1136/jmg-2022-108740

[20] Roberts ME Ranola JMO Marshall ML : Comparison of CDH1 penetrance estimates in clinically ascertained families vs families ascertained for multiple gastric cancers. JAMA Oncol 5:1325, 2019 31246251 10.1001/jamaoncol.2019.1208 PMC6604087

[21] Xicola RM Li S Rodriguez N : Clinical features and cancer risk in families with pathogenic CDH1 variants irrespective of clinical criteria. J Med Genet 56:838-843, 2019 31296550 10.1136/jmedgenet-2019-105991

[22] Lee CYC Olivier A Honing J : Endoscopic surveillance with systematic random biopsy for the early diagnosis of hereditary diffuse gastric cancer: A prospective 16-year longitudinal cohort study. Lancet Oncol 24:107-116, 2023 36509094 10.1016/S1470-2045(22)00700-8

[23] Asif B Sarvestani AL Gamble LA : Cancer surveillance as an alternative to prophylactic total gastrectomy in hereditary diffuse gastric cancer: A prospective cohort study. Lancet Oncol 24:383-391, 2023 36990610 10.1016/S1470-2045(23)00057-8 PMC10084814

[24] Gamble LA Rossi A Fasaye GA : Association between hereditary lobular breast cancer due to CDH1 variants and gastric cancer risk. JAMA Surg 157:18-22, 2022 34643667 10.1001/jamasurg.2021.5118 PMC8515254

[25] Figueiredo J Melo S Carneiro P : Clinical spectrum and pleiotropic nature of CDH1 germline mutations. J Med Genet 56:199-208, 2019 30661051 10.1136/jmedgenet-2018-105807 PMC6581119

[26] Vos EL Salo-Mullen EE Tang LH : Indications for total gastrectomy in CDH1 mutation carriers and outcomes of risk-reducing minimally invasive and open gastrectomies. JAMA Surg 155:1050-1057, 2020 32997132 10.1001/jamasurg.2020.3356 PMC7527942

[27] Kaurah P Talhouk A MacMillan A : Hereditary diffuse gastric cancer: Cancer risk and the personal cost of preventive surgery. Fam Cancer 18:429-438, 2019 31273560 10.1007/s10689-019-00133-9 PMC8164729

[28] Strong VE Gholami S Shah MA : Total gastrectomy for hereditary diffuse gastric cancer at a single center: Postsurgical outcomes in 41 patients. Ann Surg 266:1006-1012, 2017 27759617 10.1097/SLA.0000000000002030

[29] Dindo D Demartines N Clavien PA: Classification of surgical complications: A new proposal with evaluation in a cohort of 6336 patients and results of a survey. Ann Surg 240:205-213, 2004 15273542 10.1097/01.sla.0000133083.54934.ae PMC1360123

[30] Ameli R Sinaii N Luna MJ : The National Institutes of Health measure of Healing Experience of All Life Stressors (NIH-HEALS): Factor analysis and validation. PLoS One 13:e0207820, 2018 30540764 10.1371/journal.pone.0207820 PMC6291293

[31] Garland SN Pelletier G Lawe A : Prospective evaluation of the reliability, validity, and minimally important difference of the Functional Assessment of Cancer Therapy-Gastric (FACT-Ga) quality-of-life instrument. Cancer 117:1302-1312, 2011 20960518 10.1002/cncr.25556

[32] Cella D Eton DT Lai JS : Combining anchor and distribution-based methods to derive minimal clinically important differences on the Functional Assessment of Cancer Therapy (FACT) anemia and fatigue scales. J Pain Symptom Manage 24:547-561, 2002 12551804 10.1016/s0885-3924(02)00529-8

[33] Davis JL Ripley RT: Postgastrectomy syndromes and nutritional considerations following gastric surgery. Surg Clin North Am 97:277-293, 2017 28325187 10.1016/j.suc.2016.11.005

[34] Schwizer W Thumshirn M Dent J : Helicobacter pylori and symptomatic relapse of gastro-oesophageal reflux disease: A randomised controlled trial. Lancet 357:1738-1742, 2001 11403809 10.1016/S0140-6736(00)04894-7

[35] Armstrong D Bennett JR Blum AL : The endoscopic assessment of esophagitis: A progress report on observer agreement. Gastroenterology 111:85-92, 1996 8698230 10.1053/gast.1996.v111.pm8698230

[36] Sneineh MA Harel L Elnasasra A : Increased incidence of symptomatic cholelithiasis after bariatric Roux-en-Y gastric bypass and previous bariatric surgery: A single center experience. Obes Surg 30:846-850, 2020 31901127 10.1007/s11695-019-04366-6

[37] Belgau I Johnsen G Græslie H : Frequency of cholelithiasis in need of surgical or endoscopic treatment a decade or more after Roux-en-Y gastric bypass. Surg Endosc 37:1349-1356, 2023 36203112 10.1007/s00464-022-09676-y PMC9944031

[38] Canales BK Hatch M: Kidney stone incidence and metabolic urinary changes after modern bariatric surgery: Review of clinical studies, experimental models, and prevention strategies. Surg Obes Relat Dis 10:734-742, 2014 24969092 10.1016/j.soard.2014.03.026 PMC4167184

[39] Majumder MA Guerrini CJ McGuire AL: Direct-to-consumer genetic testing: Value and risk. Annu Rev Med 72:151-166, 2021 32735764 10.1146/annurev-med-070119-114727

[40] Sao Jose C Garcia-Pelaez J Ferreira M : Combined loss of CDH1 and downstream regulatory sequences drive early-onset diffuse gastric cancer and increase penetrance of hereditary diffuse gastric cancer. Gastric Cancer 26:653-666, 2023 37249750 10.1007/s10120-023-01395-0 PMC10361908

[41] Gullo I van der Post RS Carneiro F: Recent advances in the pathology of heritable gastric cancer syndromes. Histopathology 78:125-147, 2021 33382491 10.1111/his.14228

[42] Muir J Aronson M Esplen MJ : Prophylactic total gastrectomy: A prospective cohort study of long-term impact on quality of life. J Gastrointest Surg 20:1950-1958, 2016 27752808 10.1007/s11605-016-3287-8

[43] Worster E Liu X Richardson S : The impact of prophylactic total gastrectomy on health-related quality of life: A prospective cohort study. Ann Surg 260:87-93, 2014 24424140 10.1097/SLA.0000000000000446

[44] Hallowell N Lawton J Badger S : The psychosocial impact of undergoing prophylactic total gastrectomy (PTG) to manage the risk of hereditary diffuse gastric cancer (HDGC). J Genet Couns 26:752-762, 2017 27837291 10.1007/s10897-016-0045-8

[45] Gamble LA Grant RRC Samaranayake SG : Decision-making and regret in patients with germline CDH1 variants undergoing prophylactic total gastrectomy. J Med Genet 60:241-246, 2023 35817563 10.1136/jmg-2022-108733 PMC10248794

[46] Glassey R Ives A Saunders C : Decision making, psychological wellbeing and psychosocial outcomes for high risk women who choose to undergo bilateral prophylactic mastectomy—A review of the literature. Breast 28:130-135, 2016 27318167 10.1016/j.breast.2016.05.012

[47] Brandberg Y Sandelin K Erikson S : Psychological reactions, quality of life, and body image after bilateral prophylactic mastectomy in women at high risk for breast cancer: A prospective 1-year follow-up study. J Clin Oncol 26:3943-3949, 2008 18711183 10.1200/JCO.2007.13.9568

[48] Glassey R Hardcastle SJ O'Connor M : Perceived influence of psychological consultation on psychological well-being, body image, and intimacy following bilateral prophylactic mastectomy: A qualitative analysis. Psychooncology 27:633-639, 2018 28945295 10.1002/pon.4558

[49] Torrisi C:Body image in BRCA-positive young women following bilateral risk-reducing mastectomy: A review of the literature. Front Psychol 12:778484, 2021 34975666 10.3389/fpsyg.2021.778484 PMC8716694

